# Lateral assembly of oxidized graphene flakes into large-scale transparent conductive thin films with a three-dimensional surfactant 4-sulfocalix[4]arene

**DOI:** 10.1038/srep10716

**Published:** 2015-06-04

**Authors:** Ashok K. Sundramoorthy, Yilei Wang, Jing Wang, Jianfei Che, Ya Xuan Thong, Albert Chee W. Lu, Mary B. Chan-Park

**Affiliations:** 1 School of Chemical and Biomedical Engineering, Nanyang Technological University, Singapore 637459, Singapore; 2 Singapore Institute of Manufacturing Technology (SIMTech), 71 Nanyang Drive, Singapore 638075, Singapore; 3 Key Laboratory of Soft Chemistry and Functional Materials, Ministry of Education, Nanjing University of Science and Technology, Nanjing, P.R. China

## Abstract

Graphene is a promising candidate material for transparent conductive films because of its excellent conductivity and one-carbon-atom thickness. Graphene oxide flakes prepared by Hummers method are typically several microns in size and must be pieced together in order to create macroscopic films. We report a macro-scale thin film fabrication method which employs a three-dimensional (3-D) surfactant, 4-sulfocalix[4]arene (SCX), as a lateral aggregating agent. After electrochemical exfoliation, the partially oxidized graphene (oGr) flakes are dispersed with SCX. The SCX forms micelles, which adsorb on the oGr flakes to enhance their dispersion, also promote aggregation into large-scale thin films under vacuum filtration. A thin oGr/SCX film can be shaved off from the aggregated oGr/SCX cake by immersing the cake in water. The oGr/SCX thin-film floating on the water can be subsequently lifted from the water surface with a substrate. The reduced oGr (red-oGr) films can be as thin as 10−20 nm with a transparency of >90% and sheet resistance of 890 ± 47 kΩ/sq. This method of electrochemical exfoliation followed by SCX-assisted suspension and hydrazine reduction, avoids using large amounts of strong acid (unlike Hummers method), is relatively simple and can easily form a large scale conductive and transparent film from oGr/SCX suspension.

Transparent conductors are needed in a myriad of applications such as video displays, solar cells, lasers, organic light-emitting diodes (OLEDs), thin-film transistors (TFTs) and sensors^1^,^2^,^3^. Indium tin oxide (ITO) is one of the most widely used transparent conductors, with sheet resistance as low as ~10  Ω/sq and transparency at 550 nm higher than 85%^4^,^5^. ITO applications are limited (particularly in flexible devices) due to brittleness, cost, and scarcity. As a result, research aimed at finding alternative materials has been conducted^6^,^7^,^8^,^9^. Various candidate materials have been investigated, such as transparent and conductive zinc oxide films (ZnO:Al and ZnO:Ga), TiO_2_:Nb films, silver nanowire, conductive polymers, and carbon nanotubes and few-layers graphene (FLG) intercalated with ferric chloride^10^,^11^,^12^,^13^,^14^,^15^,^16^.

Graphene has also been considered a strong contender for use as a transparent conductor^17^. Many methods have been reported for the fabrication of graphene films, including ultra-high vacuum chemical vapor deposition (CVD), deposition of single- to few layers graphene films on polycrystalline Ni by CVD (sheet resistance = 770−1000 Ω/sq), Langmuir-Blodgett (LB), vacuum filtration, evaporation-induced self-assembly, spray-coating and spin-coating^18^,^19^,^20^,^21^,^22^,^23^. Among these methods, solution processing of few-layers graphene (FLG) is economical in addition to achieving a transparency comparable to the thin films prepared by more complicated methods such as LB. Common solution processing methods such as spray-coating and spin-coating usually result in deposition of fairly thick films; these approaches achieve continuity between the FLG islands but at the cost of reduced transparency^17^,^24^. Furthermore, some of these methods (*e.g.* evaporation-induced self-assembly, which requires heating) are usually not applicable for flexible substrates. Hence, there is great need for new methods that can produce continuous thin film easily, but without the need for high temperature treatment, high vacuum, etching or organic solvents.

Due to their unique cup-shape structure, amphiphilic calix[n]arene molecules with different functional groups can form various self-assembly structures, including nanocapsules, micelles and bilayers in aqueous solution^25^,^26^,^27^. The family of calix[n]arenes, which consists of a repeating phenolic unit formed into a macrocycle via methylene bridges, has been shown to facilitate self-assembly of cobalt and gold nanoparticles, but not yet graphene or FLG^28^,^29^. The amphiphilic calix[n]arene molecule 4-sulfocalix[4]arene sodium salt (SCX) (Fig. 1A) has been widely studied as a coupling agent for organic molecules and metal complexes. SCX provides both hydrophobic (aromatic ring) and hydrophilic (SO_3_^−^) environments, similar to surfactants and micelles^30^,^31^,^32^. It is known that SCX can form a dimeric supramolecular nanochannel/nanopore-like structure through charge-assisted π-stacking interaction with metal complexes (M^2+^ and M^3+^). Zheng and co-workers observed that [Tb(2,2’-bipyridine-N,N’-dioxide, bpdo)_4_]^3+^ and SCX can form a layered structure, in which the calix[4]arenes are in a bilayer arrangement^33^,^34^.

Herein, we report a new and simple solution-based method for lateral assembly of small few-layers partially oxidized graphene (oGr) flakes into macroscopic ultrathin conductive films on various substrates via a 3-step process (Fig. 1B). The oGr flakes were first synthesized by electrochemical exfoliation, followed by washing and dispersion in water with the novel three-dimensional (3-D) surfactant SCX. The single- to few-layers-thick oGr flakes were characterized by Atomic Force Microscopy (AFM) and Raman spectroscopy. To assemble the oGr flakes, a three-step process involving filtration, dipping the filtration cake into water and fishing out the thin shaved-off layer from the water surface is employed (Fig. 1B). First, the oGr/SCX was deposited on a filter membrane by vacuum filtration of the suspension. Second, the oGr/SCX cake on the filter membrane was immersed in water, resulting in the “shaving” off of an ultra-thin layer from the top of the oGr/SCX cake; leaving behind a fairly thick layer of oGr/SCX on the filter membrane. Third, the floating oGr/SCX thin film was “fished” out from the surface of the water and onto the desired substrate. This procedure may be repeated to fabricate multiple oGr/SCX thin films from the same oGr/SCX cake. After the transfer onto a substrate, the oGr film was reduced by immersion into hydrazine solution in order to decrease sheet resistivity. The resulting continuous red-oGr thin film obtained was shown to be highly transparent and of low electrical resistance. We have successfully assembled continuous thin (10−20 nm) films of red-oGr with a lateral dimension of 25 mm, transparency of >90% and sheet resistance of 890 ± 47 kΩ/sq on glass slides.

## Results and Discussion

Few-layers oGr flakes were synthesized by electrochemical exfoliation of graphite sheet in a single compartment electrochemical cell in the presence of Li^+^ ions at high voltage by the intercalation mechanism^35^,^36^. During the exfoliation, Li^+^ ions were reversibly intercalated into the graphite interlayers in a series of well-defined stages^37^. Figure 2A shows the Raman spectra of graphite, oGr/SCX and red-oGr. According to the literature, when the intensity of the 2D band is higher than or equal to that of the G band, the graphene is respectively monolayer or bilayer thick^38^,^39^. The observed 2D to G bands intensity ratio (1:6) implies that our oGr film is composed of a few layers. The spectrum of oGr/SCX film (Fig. 2A, line ii) also exhibits a strong D band at 1353 cm^−1^, which is absent in the graphite spectrum (Fig. 2A, line i). This new D spectral feature in the exfoliated oGr, with an I_D_/I_G_ intensity ratio of 1.26, is attributable to an increase in the defect density in the graphitic structure due to electrochemical exfoliation; pristine graphite has a I_D_/I_G _= ~0. Due to the oxidation of the exfoliated oGr, a reduction step is added to restore graphene-like electrical properties in the thin film; this was accomplished with the hydrazine solution treatment at 70 °C. In the Raman spectrum of the resulting red-oGr, the D band was significantly increased so that I_D_/I_G_ is now 1.65 due to restored *sp*^2^-hybridized carbons from reduction of oxygenated C atoms (COOH, epoxide *etc.*) (Fig. 2A, line iii). This increased I_D_/I_G_ ratio of red-oGr film could be related to the re-established graphene network which has a smaller average size *sp*^2^ domain than the original oGr film. This observation was previously reported for chemically reduced GO films^40^,^41^. In other words, the average size of the *sp*^2^ domain in oGr decreased after hydrazine reduction, which could lead to the increase in I_D_/I_G_ ratio. Our results suggested that the partially oxidized groups of oGr might be reduced by hydrazine treatment^40^,^42^,^43^.

X-ray photoelectron spectroscopy (XPS) analysis was used to probe the functional groups on the various samples. The XPS wide scan showed the incorporation of increased amounts of O1s and small amounts of sulphur in the exfoliated oGr/SCX flakes compared to graphite (Table 1); these are respectively attributed to partial oxidation and the presence of the SCX surfactant (Table 1). The high resolution C1s spectrum of graphite showed peaks at 284.5 and 285.6 eV which could be assigned to the non-oxidized ring C-C atoms, and C-C atoms in defective structure of graphite, respectively (Fig. 2B). However, the high resolution C1s spectrum of oGr showed peak at 284.5, 285.5, 286.8, and 288.5 eV which were assigned to the non-oxidized ring C-C atoms, C-C atoms in defective structure of graphene lattice, C atoms bonded to hydroxyl/epoxy/ether functional groups, and the C atoms in carbonyl groups, respectively (Fig. 2C)^44^,^45^. This result clearly demonstrated that as-produced oGr had considerable degree of oxidation with residual SCX molecules (Table 1 and Fig. 2B,C).

As-prepared oGr flakes in water readily aggregate and stack in multi-layers due to van der Waals interactions. These aggregates usually have inferior physical and chemical properties compared to those in the exfoliated monolayer state^46^,^47^. As such, SCX (which acts as a surfactant to limit aggregation) was added to the oGr solution and the resulting suspension was ultrasonicated and then centrifuged. Photographs of both oGr/SCX dispersion and oGr-water (control) were taken immediately after preparation and after standing for 24 hrs at room temperature. The oGr/SCX dispersion (Fig. 3A(i)) showed no evidence of aggregation even after standing (at room temperature) for more than four weeks. In contrast, in the absence of SCX, the oGr flakes aggregated and settled (Fig. 3A(ii)). We hypothesize that the SCX stabilizes the oGr either through π-π interaction or electrostatic interactions/charge transfer^48^. Residual Li^+^ on the oGr flake surfaces may interact electrostatically with the sulfonic groups and via charge transfer with the hydroxyl groups of SCX.

In the films prepared from the oGr/SCX dispersion, the diameters of the oGr flakes were measured by AFM to be 0.3–1.7 μm, with an average thicknesses of 10 nm (Fig. 3B(i))^49^. For the oGr/SCX dispersion, we also evaluated the particle size by dynamic light scattering. Figure 3C(i) show plots of intensity against diameter distribution for the SCX solution (0.5 mM) and oGr/SCX dispersions. Results show that the SCX micelle diameter in aqueous solution to be around 200 nm. For the oGr/SCX dispersion, the diameter of the composite oGr/SCX flakes is in the range of 0.8–1.8 μm, which is in agreement with the AFM results (Fig. 3B ii).

To further confirm the presence of SCX on the oGr flakes, energy dispersive X-ray spectroscopy (EDX) was used. Significant amounts of sulphur (S) and sodium (Na) in oGr/SCX observed, provides evidence of the strong binding of the SCX molecules to oGr (Fig. 4A, Table 2 and Fig. S1). Additionally, thermal degradation plots of oGr/SCX in air showed two major degradation weight losses from 305–475 °C (~53%) and from 475–650 °C (~46%) corresponding respectively to SCX (denoted as region I in Fig. 4B) and oGr (denoted as region II). The SCX powder itself has about 10% weight loss below 200 °C most likely due to moisture desorption and a significant weight loss of about 50% from 330 to 507 °C (region I); the residual mass of about 40% at temperatures above 507 °C is attributed to the sodium content of SCX (Fig. 4B, line i). In the pristine oGr flakes, two major weight loss features of about 24% from 360–460 °C and about 61% from 470–660 °C (region II), were observed and were attributed respectively to the decomposition of the dangling oxygenated compounds and the graphene itself ((Fig.4B, line iii). The TGA plot of oGr/SCX shows approximately a 1:1 weight ratio of oGr to SCX (Fig.4B, line ii) which indicated that there was higher surface coverage of SCX on oGr flakes^50^,^51^.

**Figure 1:**
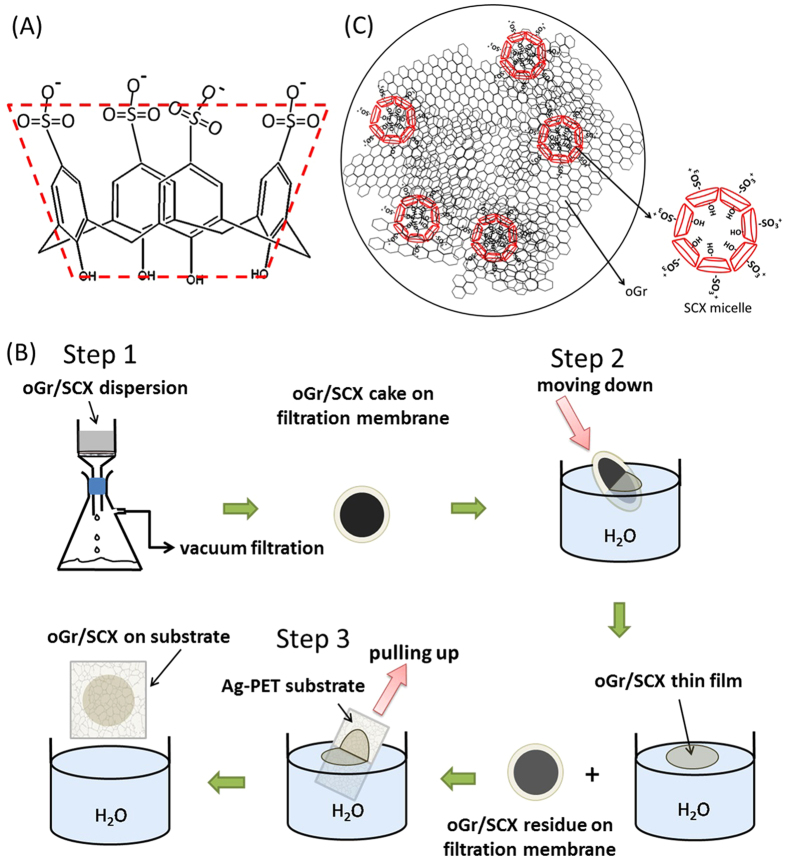
(**A**) 3-D structural formula of SCX. (**B**) A schematic representation of the oGr/SCX film transfer onto a substrate. (**C**) The proposed action of SCX to promote self-assembly of the oGr/SCX film.

**Figure 2:**
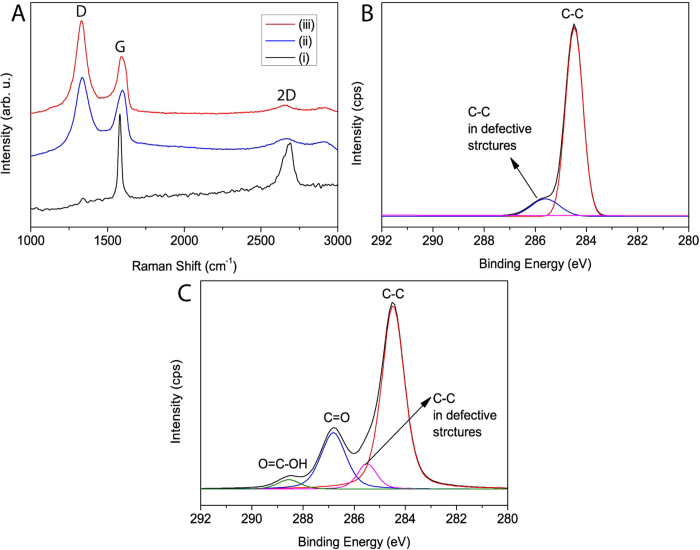
(**A**) Raman spectra of (i) Graphite, (ii) oGr/SCX film and (iii) red-oGr film. XPS high resolution C1s peak deconvolution data of (**B**) graphite sheet and (**C**) oGr flakes.

**Figure 3:**
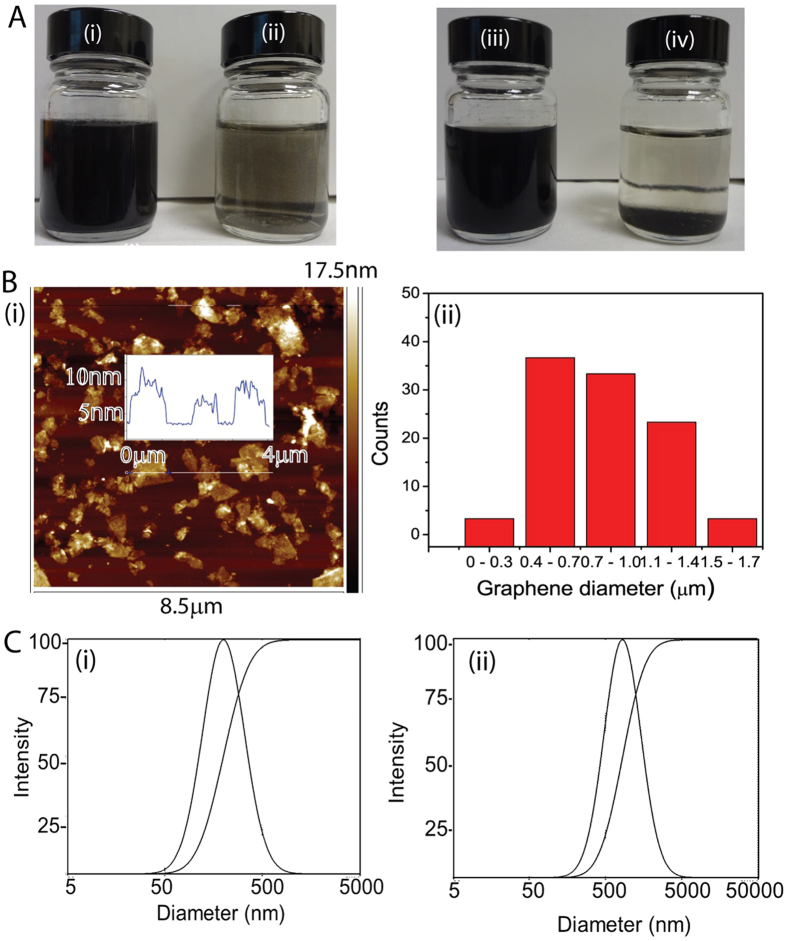
(**A**) Images of as-synthesized (i, iii) oGr/SCX and (ii, iv) oGr/water dispersions before (i, ii) and after standing (iii, iv) at room temperature for 24 h. (**B**) (i) Height and (ii) diameter measurements of the oGr flakes by AFM. (**C**) Dynamic light scattering data of size distributions of (i) SCX in water and (ii) oGr/SCX dispersion.

**Figure 4:**
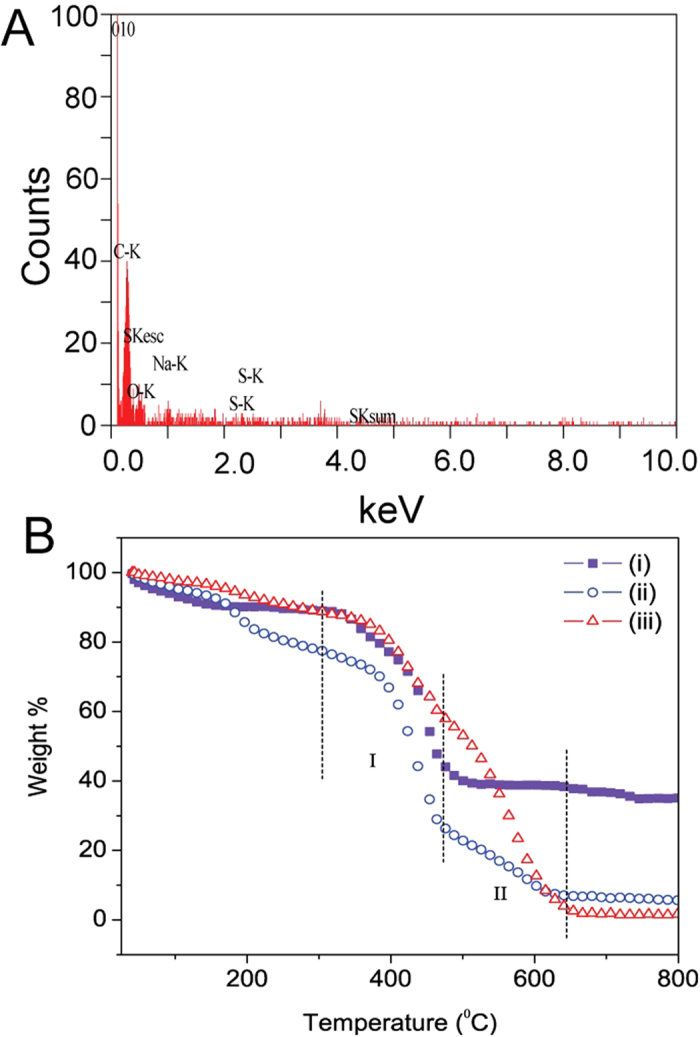
(**A**) EDX spectrum of the oGr/SCX flakes. (**B**) TGA measurements in air of (i) pure SCX, (ii) oGr/SCX flakes and (iii) oGr flakes (Temperature scan rate is 10 °C/min.)

**Figure 5:**
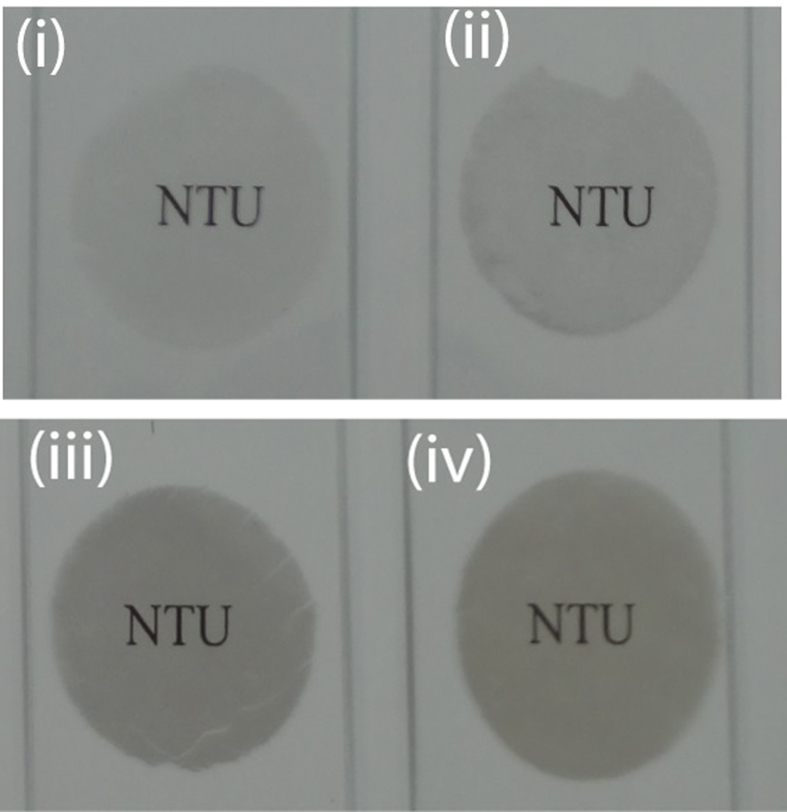
Photographic images (i-iv) of the red-oGr films (of increasing thickness) deposited on transparent glass slides.

The oGr/SCX dispersion may be used to make transparent and conductive red-oGr thin films. Figure 1B schematically illustrates the oGr/SCX film transfer onto substrates. oGr flakes dispersed in SCX aqueous solution (0.5 mM) is shown in Fig. S2. With vacuum filtration, a thick cake of oGr/SCX was formed on the filter membrane. Next, the filter membrane with the wet oGr/SCX cake was slowly inserted into the water (at ~45 °C). A thin film of oGr/SCX would then be naturally peeled from the oGr/SCX cake and float on the surface of the water (Supplementary video). Using a substrate, we collected the floating oGr/SCX thin film from the water by immersing the substrate into the water, aligning it with the oGr/SCX film, and lifting the thin film out of the water (Fig. 1B, Step 3). The thin oGr/SCX film could easily be transferred (without cracking) from the water onto the substrate even with our hand assisted transfer method (Supplementary video). By adjusting the volume of the oGr/SCX solution used during vacuum filtration, the thickness of the wet oGr/SCX film on the filter membrane and the thickness of the final peeled/deposited oGr/SCX film could be adjusted. After hydrazine reduction at 70 °C for 45 min, resistivity measurements were carried out on the red-oGr films (of different thicknesses) deposited on the glass slides (Fig. 5). Table 3 shows the sheet resistances and transparencies (recorded at 550 nm) of the red-oGr films (Fig. S3). With a film thickness of >200 nm (at T < 80%), we can achieve sheet resistance of about 56.5 ± 19.1 kΩ/sq. With thinner samples of >90% transmittance, the resistance is 890.0 ± 47.0 kΩ/sq. Our results are comparable with the outstanding results of others using solution-based transfer method to achieve reduced GO film with resistivity of ~150 kΩ/sq at T_550_ = 80% (thicker sample) or resistivity of 20 ΜΩ/sq at T _550_ = 95%^52^. In that work, the transferring stamp was a mixed cellulose ester membrane that needed to be dissolved away by acetone after the transfer; however, our method does not require this “clean-up” step. The sheet resistivity of red-oGr film was still higher than other graphene based transparent films;^16^,^53^,^54^ however, these reported transparent graphene films (in small scale) were prepared on a small substrate/device by CVD methods with/or without high temperature annealing/vacuum. Our oGr film transfer method was entirely based on a solution process, and large-area oGr film could be transferred onto both rigid and flexible substrates by simple hand assisted transfer method without performing tedious transfer/chemical etching/annealing processes. It is possible to further improve the electrical properties of red-oGr film by using hybrid nanomaterials, such as silver nanowires or metallic single-walled carbon nanotubes^55^,^56^.

This is the first report on the lateral assembly of oGr flakes into a fairly thin macroscopic oGr film with the use of the unique SCX surfactant. In our process, we used electrochemical exfoliation and the SCX surfactant for the preparation of stable oGr dispersion. Our process avoids the need to use highly oxidized GO for good dispersibility. The typical oxidation of graphite powder into GO sheets (using Hummers method) is a dangerous process and generates a lot of waste due to the use of large quantities of concentrated acid (H_2_SO_4_) and strong oxidizing agent (KMnO_4_). This process usually takes several days to finish and the resultant GO sheets are completely isolated. Our electrochemical exfoliation-SCX-hydrazine reduction process is significantly faster, generates less waste, and can be easily integrated into a solution processing line.

It is typically difficult to make a continuous thin film from GO flakes in order to achieve good conductance and high transparency over a large area, a common method used is transfer printing. Other feasible solution processing methods include repeated dip-coating, and spray drying. In the transfer printing of GO thin film, the process of complete pick-up and deposition on the target substrate is rather tricky or messy and could result in a discontinuous film or the need for large amounts of solvent. On the other hand, repeated dip-coating is laborious, while for spray drying, it is typically difficult to control the film thickness. We report here an alternative to transfer printing in making thin oGr films by simply using a self-assembling 3-D surfactant. Furthermore, our process does not require high-temperature thermal reduction because our oGr is still somewhat conductive, and is also organic solvent-free.

**Figure 6:**
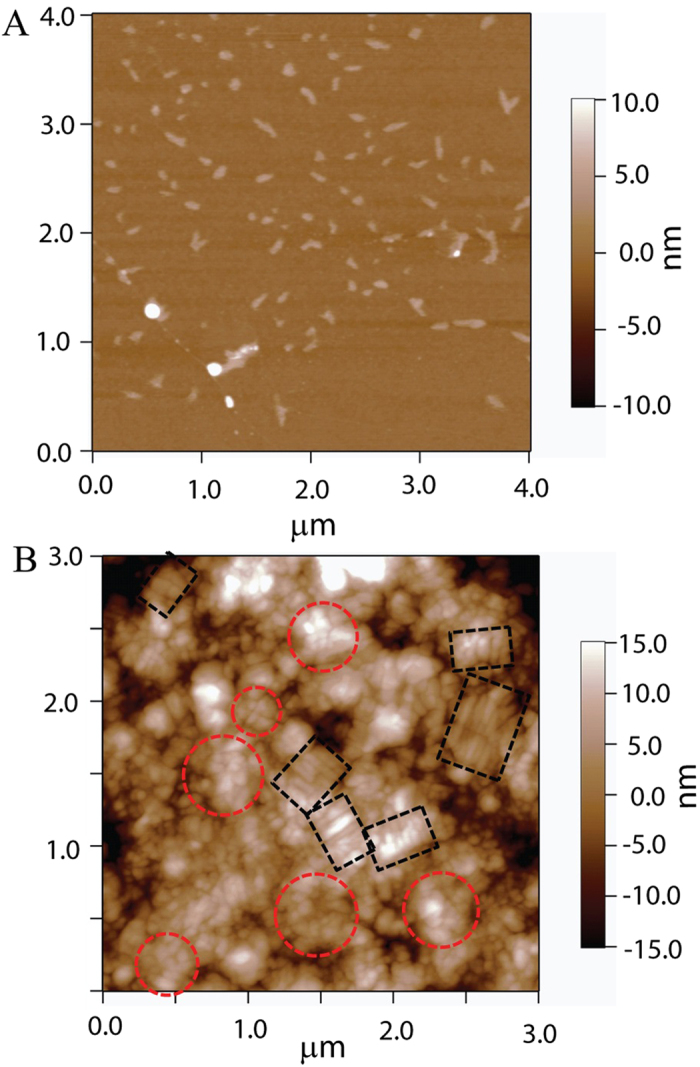
(**A**) AFM image of the SCX micelles (~100 nm across and 2−4 nm in height) dried on the silicon wafer surface. (**B**) AFM image of assembled SCX on the oGr flakes dried on the silicon wafer surface. Parallel aligned cylindrical structures (~100 nm across and 8−10 nm in height) and disk structures (~100 nm diameter and 10 nm in height) are highlighted by black boxes and red circles, respectively.

To investigate the morphologies of SCX on the oGr/SCX films, we performed AFM studies of SCX on wafer and also on the oGr/SCX surfaces. Figure 6A shows the topography of SCX after depositing and drying the SCX solution (0.5 mM) on a silicon wafer surface. The AFM image confirms the micelle formation of SCX in aqueous solution, as seen from the uniform individual aggregates ~100 nm across and 2–4 nm thick, on the silicon wafer surface. Similar results have been reported elsewhere^57^. Dried oGr/SCX film on the silicon wafer surface exhibits side-by-side cylindrical structures (boxes, Fig. 6B) as well as circular clusters (circles, Fig. 6B). When the oGr flakes are sonicated with SCX in aqueous solution, the SCX molecules assemble onto the oGr particles due to hydrophobic interactions between the SCX sidewall and the oGr surface; while their hydrophilic ends interact with other SCX molecules. We hypothesize that these unique structures may “gel” two oGr flakes during the filtration process (Fig. 1B, Step 1) and assist in the assembly of the oGr flakes into a large thin film. In addition to this “gelation” mechanism, the strong hydrophobic interaction between two oGr/SCX flake areas that are not covered by the surfactant micelles may be another “gelation” mechanism.

To further investigate the mechanism of the lateral assembly of the oGr/SCX, we performed control studies by dispersing oGr flakes in other surfactants such as sodium cholate (SC), naphthalene sulfonic acid (NSA), and in the organic solvents (such as dimethylformamide (DMF) and tetrahydrofuran (THF)). We found that SCX is a better dispersant of oGr than SC, and electrochemically exfoliated oGr flakes are not soluble in THF. The oGr/SCX dispersion remained stable and without aggregation for more than four weeks at room temperature (Fig. S4)^48^. With both the oGr/SC and oGr/NSA solutions, the oGr film did not peel off from the filtration cake on the filter membrane upon immersion in water (Fig. S5).

**Figure 7:**
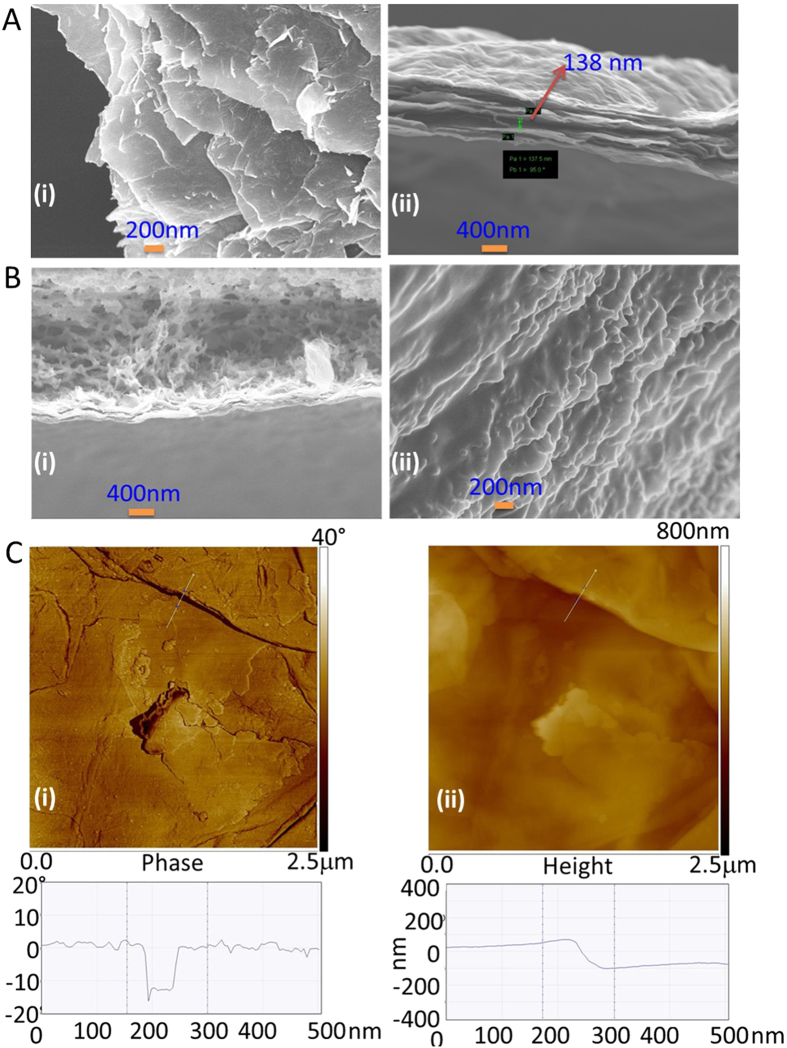
(**A**) FESEM of oGr/SCX cake (top view (i) and cross-sectional view (ii)). (**B**) FESEM of oGr/SC cake (top view (i) and cross-sectional view (ii)). (**C**) AFM phase (i) and height measurements (ii) of the oGr/SCX cake. For these measurements, the oGr flakes were dispersed separately in SCX (0.5 mM) and SC solutions (0.5 mM). Both the oGr/SCX and the oGr/SC cakes were obtained by vacuum filtration on membranes. Finally, both the oGr/SCX and the oGr/SC cakes were dried and broken to view the top and cross-section of the films under FESEM and AFM.

Figure 7A,B present the cross-sectional views (acquired by field emission scanning electron microscopy (FESEM)) of the thick oGr/SCX (Fig. 7A images i and ii) and oGr/SC (Fig. 7B images i and ii) cakes on the filter membranes. The layered structure and interlayer gaps on the oGr/SCX cross-section (Fig. 7A(i,ii)) are plainly visible. A similarly prepared film obtained from the oGr/SC suspension exhibits no layers or inter-layer gaps (Fig. 7B(i,ii)). For the oGr/SCX, there are layers of oGr sheets stacked in a step-wise configuration (Fig. 7A(i)). Using SEM, the layer step heights in the oGr/SCX were measured to be 100-138 nm (Fig. 7A image ii), corroborating the AFM results (Fig. 7C(i)). In contrast, for the oGr/SC films, the layers of graphene are more closely packed (Fig. 7B(i,ii)). It is probably because of this close packing of the oGr layers with the SC surfactant that result in the inability of the thin oGr film to peel from the wet cake on the filter membrane, when placed under aqueous immersion. The 3-D SCX surfactant prevents the oGr flakes from being closely adhered to one another and facilitates the delamination in water. At the same time, their amphiphilic character may result in micelle formation that “gels” two oGr flakes together.

Generally, compounds such as sulfonic acids and aryl carboxylic acids, are good dopants and could enhance the conductivity of the materials after doping^58^,^59^,^60^. Due to the presence of four sulfonic acid groups on the polycyclic ring in SCX (Fig. 1A), SCX is expected to be a good doping agent for the oGr/SCX films^59^. Using SCX as a dispersing agent, we achieved a stable oGr/SCX suspension with π-π stacking or charge transfer; in addition to the prevention of van der Waals-driven aggregation of the oGr flakes by the SCX molecules. To the best of the authors’ knowledge, this is the first report on the use of SCX as a dispersant which enables film formation and transfer without significant conductivity-degrading contamination on the oGr film.

**Figure 8:**
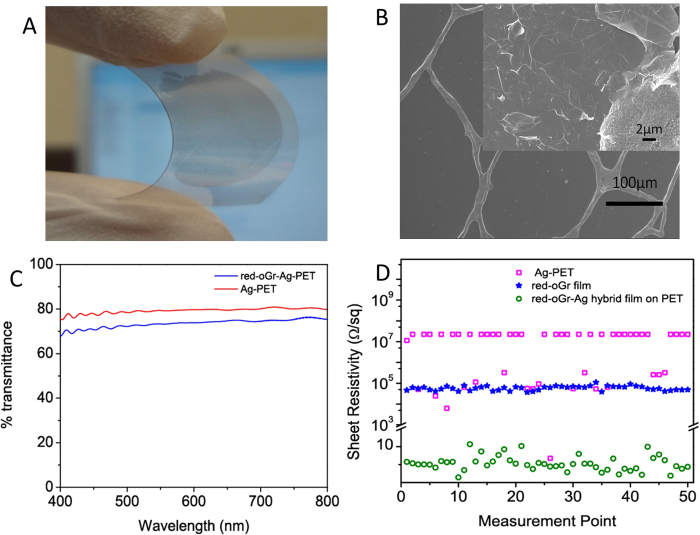
(**A**) Images of the red-oGr film coated Ag-grid PET substrate (dark circle indicates red-oGr film covered area). (**B**) FESEM image of the bare Ag-grid PET substrate. (Inset is the magnified FESEM image of the oGr/SCX film on Ag-grid.) (**C**) The transmittance spectra recorded before (red curve) and after coating (blue curve) of red-oGr film on Ag-grid PET. (**D**) Sheet resistances of bare Ag-PET (squares), red-oGr film (stars) and red-oGr-Ag-PET film (circles) (Sheet resistance could not be measured all over Ag-PET (squares) without coating of red-oGr which implied that sheet resistance has been significantly improved upon coverage of transparent red-oGr film, green circles).

To explore the possibilities for alternative substrates (Fig. 1B, Step 3), and also to prepare flexible transparent thin films with much lower resistivity, the oGr/SCX film was deposited onto commercially available Ag-PET film, which has an irregular grid of Ag lines on the PET plastic substrate (Fig. 8A,B). The grid is discontinuous, with the insulating PET between the Ag lines (Fig. 8B). The oGr film can be observed clearly on top of the latticed Ag nanoparticles as well as the empty areas on the PET substrate. The red-oGr film adheres very well to the PET substrate (Fig. 8B inset). As shown in Fig. 8C, the transmittance of this hybrid film is 73.5% at 550 nm wavelength and the sheet resistance becomes much more homogeneous with the red-oGr overlayer (Fig. 8D). In addition to providing the bulk of the conductivity, the red-oGr layer acts as an oxidation and sulfidation resistance layer to protect the latticed Ag nanoparticles.

In summary, we have developed a new, efficient and organic solvent-free method to deposit oGr/SCX thin film on solid and flexible substrates with good transparency and low sheet resistance. The SCX molecules act as surfactants, promoting the lateral assembly of the oGr/SCX flakes and preventing the oGr flakes from vertically consolidating into a cohesive thick film when the suspension is filtered. When a thick cake of the oGr/SCX hybrid is immersed in water at an angle, laterally aggregated thin films of oGr/SCX peel off from the surface of the cake but retain their mechanical continuity. These thin layers have improved transparencies compared to the thick films produced by other methods; we can achieve thin films down to 10−20 nm thick with a transparency of >90% and sheet resistance of 890 ± 47 kΩ/sq, which is comparable to reduced GO (produced by Hummers method) film prepared by transfer printing. Our process does not need the highly hydrophilic GO, is entirely water-based, and does not require the excessive use of strong acids/oxidizing agents. This method is an attractive alternative to the production of continuous thin films of oGr for various applications. Furthermore, the oGr film can be transferred onto any substrate, permitting the integration of other active/compatible materials with graphene based films for advanced electronic device applications.

## Materials and Methods

### Chemical and Reagents

Graphite sheet was purchased from Graphitestore.com, Inc., IL, US. Lithium perchlorate (LiClO_4_), SCX, SC and NSA were purchased from Sigma-Aldrich. All other reagents were analytical grade unless otherwise specified.

### Preparation of oGr/SCX dispersion

First, oGr flakes were obtained from the electrochemical exfoliation of graphite^35^. For this, a single compartment electrochemical cell was employed. Graphite sheet and platinum wire were used as anode and cathode, respectively. 10 mL of 0.1 M LiClO_4_ solution was used as the electrolyte (Fig. S6). Argon gas was bubbled to remove oxygen from the electrochemical cell. The argon environment was maintained throughout the subsequent graphite exfoliation. Electrochemical exfoliation was performed for 45 mins with 15 V applied across the electrodes. After successful exfoliation of the graphite, the oGr flakes were mixed with 0.5 mM of SCX solution and bath sonicated for 30 mins, followed by tip sonication for 30 mins (Fig. S6, Step 2). The resulting transparent yellow-brown colored oGr/SCX dispersion was centrifuged at 2000 g for 30 mins. After centrifugation, the top fraction (~60%) of the supernatant solution was withdrawn from the centrifuge tubes and used for characterization studies. For control studies, similarly prepared (but without SCX) oGr flakes were dispersed in SC (0.5 mM), NSA, DMF and THF.

### Transparent oGr/SCX wet-film transfer onto solid and flexible substrates

The required volume of the oGr/SCX dispersion was diluted with water and mixed well by bath sonication. The as-diluted homogeneous oGr/SCX dispersion was filtered through 25 mm or 45 mm filter membranes (pore size ~0.2 μm) by vacuum filtration. After filtration, the wet cake of oGr/SCX on the membrane was slowly inserted into still water (insertion angle of ~45°); a thin oGr/SCX film would then peel off from the membrane and float on the water (Fig. 1B and Supplementary video). This oGr/SCX film had decent handling durability showing potential for ease of transfer onto others substrates such as glass, Si wafer, and PET films. We first transferred the oGr/SCX film with a size of about 25 mm onto solid glass substrate (see supplementary video). We also successfully transferred the oGr films with various thicknesses onto glass substrates. After successful film transfer, the oGr/SCX films were washed with water several times and annealed at ~100 °C for 1 h. We further reduced the oxidized groups of the oGr films on the substrates by dipping the oGr film into alkaline hydrazine solution at 70 °C for 45 mins. The various film thicknesses of the red-oGr films are listed in Table 3 with the optical transparency and resistivity. For a film thickness of 10−20 nm, sheet resistivity of 890 ± 47 kΩ/sq (at T > 90%) was obtained. Additionally, we successfully transferred the oGr/SCX thin films onto silver grid (Ag-grid) pre-patterned on PET film^55^. After the oGr/SCX film transfer, the red-oGr-Ag-grid-PET film was dried at 80 °C for 3 h. FESEM image of the red-oGr thin film on Ag-grid indicates that a uniform coverage of conductive film was formed.

### Equipment and characterization

DC power supply (Agilent E3645A) was used for electrochemical exfoliation. Raman spectra were measured with a Renishaw Raman scope in backscattering configuration using a 633 nm wavelength laser over graphite, oGr and red-oGr samples. Transmittance spectra were measured (400 to 800 nm) using a Varian Cary 5000 UV-VIS-NIR spectrophotometer. The surface morphology of the oGr/SCX film was measured using a scanning electron microscope (FESEM, JOEL, JSM-6700F). Atomic force microscopy (AFM) was conducted using a Dimension Icon Atomic Force Microscope (Bruker, USA) in tapping mode. Sheet resistances of the red-oGr films were measured by using a Keithley 236 Source Measure Unit with four point vacuum probe lock from Cascade Microtech Inc, OR, USA. A Fluke 45 dual display multimeter was used to measure the current. Thermo-gravimetric analysis (TGA) was performed using a Perkin Elmer TGA/DTA (Pyris Series-Diamond TG/DTA) equipment under air with a heating rate of 10 °C/min and a temperature range of 50−800 °C.

## Additional Information

**How to cite this article**: Sundramoorthy, A. K. *et al*. Lateral assembly of oxidized graphene flakes into large-scale transparent conductive thin films with a three-dimensional surfactant 4-sulfocalix[4]arene. *Sci. Rep.* **5**, 10716; doi: 10.1038/srep10716 (2015).

## Supplementary Material

Supplementary Information

Supplementary Video

## Figures and Tables

**Table 1:** Atomic concentration of carbon, oxygen and sulfur in (i) Graphite sheet and (ii) oGr/SCX flakes by XPS wide scan.

**Sample**	**C1s position (eV)**	**C1s (%)**	**O1s (%) (532.8 eV)**	**S2p (%) (169.3 eV)**
i) Graphite	284.5	97.26	2.74	—
ii) oGr/SCX	284.5	82.37	17.48	0.14

**Table 2:** Elemental composition of as-synthesized oGr flakes, SCX powder and oGr/SCX samples by EDX.

**S. No.**	**Samples**	**C (%)**	**O (%)**	**S (%)**	**Na (%)**
1.	oGr flakes	80.5	19.5	—	—
2.	SCX powder	55.6	25.4	10.5	8.5
3.	oGr/SCX flakes	77.0	20.1	1.6	1.3

**Table 3:** Sheet resistance and transparency measurements of the red-oGr thin films with different thicknesses on the glass substrates.

**Thickness (nm)**	**Transmittance (T%) measured at 550 nm.**	**Sheet Resistance (kΩ/sq)**
10-20	≥90%	890.0 ± 47.0
30-100	85%–90%	581.3 ± 169.4
100-200	80%–85%	183.4 ± 42.6
>200	<80%	56.5 ± 19.1
