# Supplementary material for: Lateral assembly of oxidized graphene flakes into large-scale transparent conductive thin films with a three-dimensional surfactant 4-sulfocalix[4]arene
Source: Sci Rep. 2015 Jun 4;5:10716. doi: 10.1038/srep10716 (PMC4455233; doi:10.1038/srep10716)
Supplement: Supplementary Information [file srep10716-s1.pdf]

(Supporting Information)

Lateral assembly of oxidized graphene flakes into large-scale transparent conductive thin films with a three-dimensional surfactant 4-sulfocalix[4]arene

Ashok K. Sundramoorthy[1* ‡ a], Yilei Wang[1‡], Jing Wang[1], Jianfei Che[3], Ya Xuan Thong[1], Albert Chee W. Lu[2] and Mary B. Chan-Park[1*]

[1]School of Chemical and Biomedical Engineering, Nanyang Technological University, Singapore 637459, Singapore

[2]Singapore Institute of Manufacturing Technology (SIMTech), 71 Nanyang Drive, Singapore 638075, Singapore

[3]Key Laboratory of Soft Chemistry and Functional Materials, Ministry of Education, Nanjing University of Science and Technology, Nanjing, P.R. China

*Correspondence and requests for materials should be addressed to M.B.C. (mbechan@ntu.edu.sg) Fax: +65 6794 7553; Tel: +65 6790 6064 or A.K.S. (ashok.sundramoorthy@wisc.edu). [a]Present address: Department of Biological Systems Engineering, University of Wisconsin-Madison, Madison, WI, United States. Tel: +1 (608) 262-1054; Fax: +1 (608) 262-1228

[‡] A.K.S. and Y.W. contributed equally to this work.

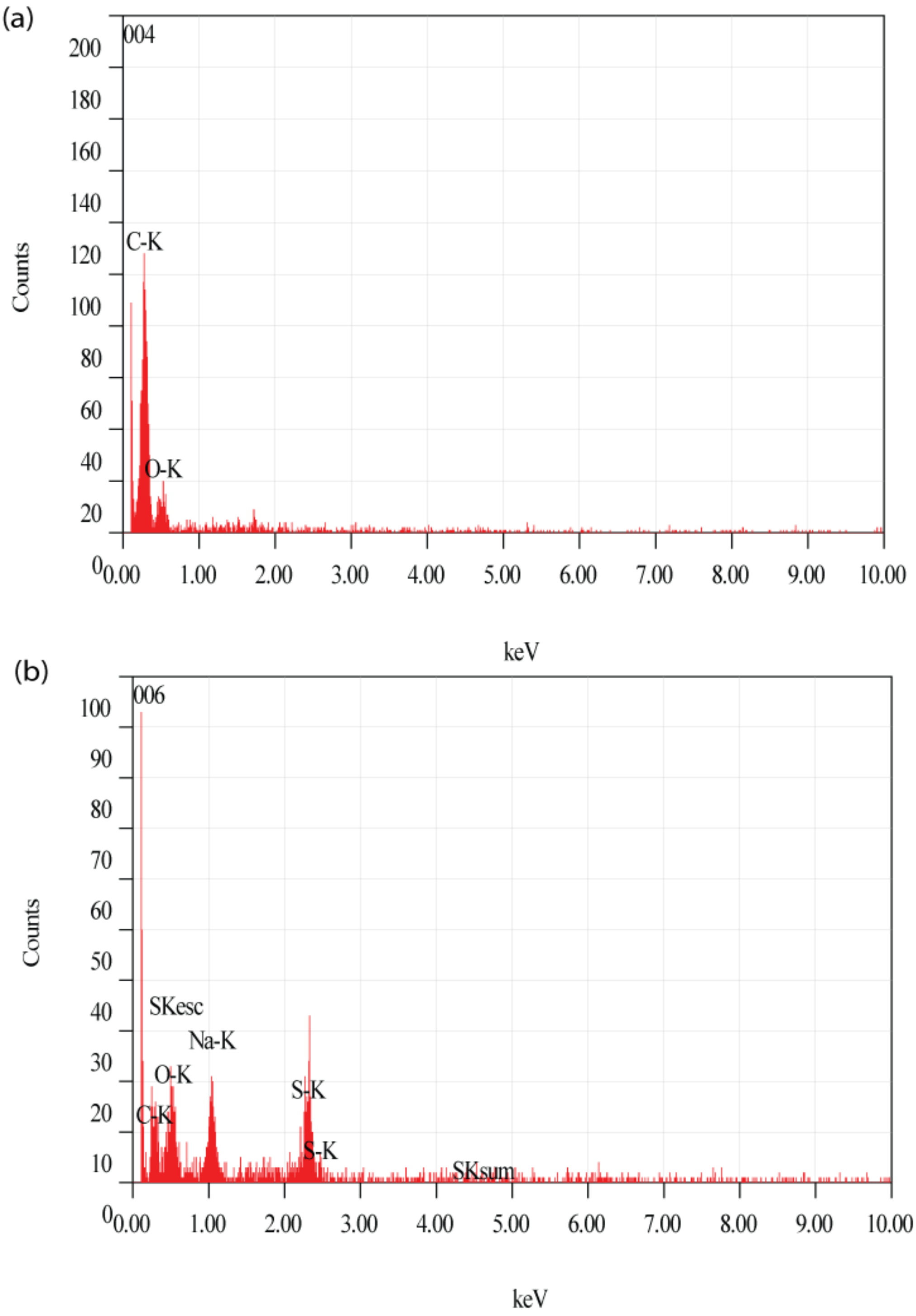

Fig. S1 EDX spectra of (a) oGr flakes (from electrochemical exfoliation) and (b) pure SCX powder

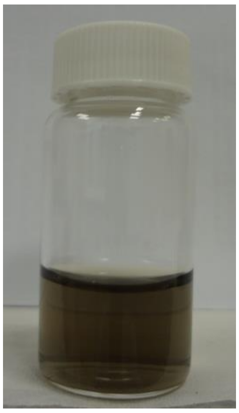

Fig. S2 oGr/SCX dispersion in water.

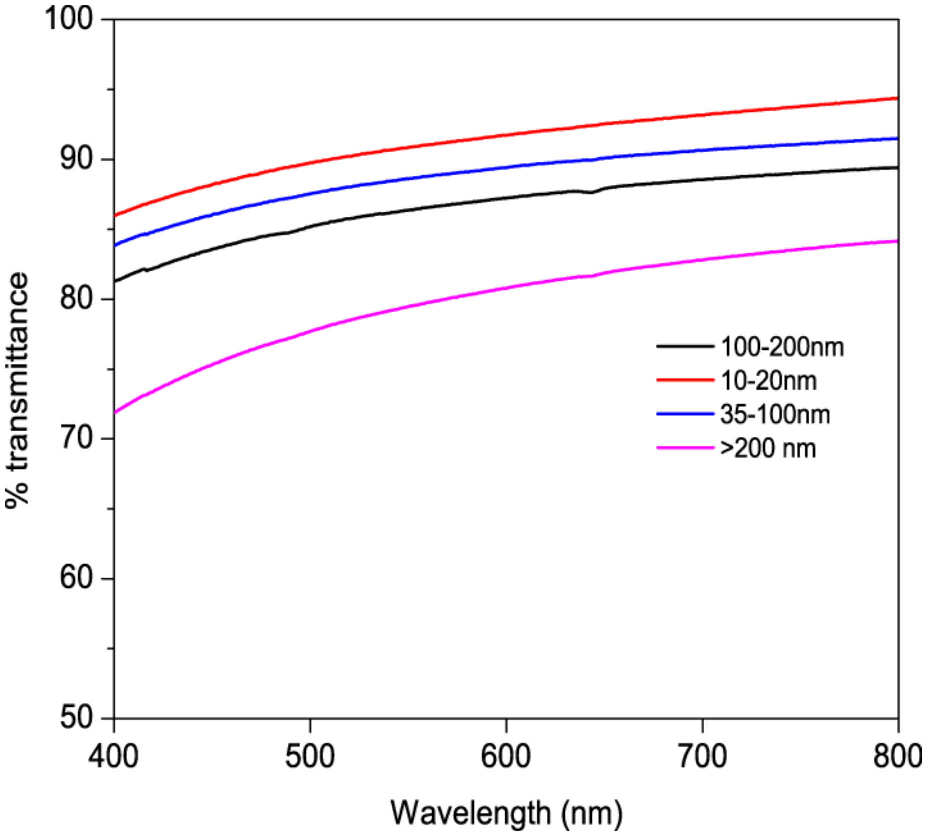

Fig. S3 The transparency measurements of the red-oGr thin films with different thicknesses on the glass substrates.

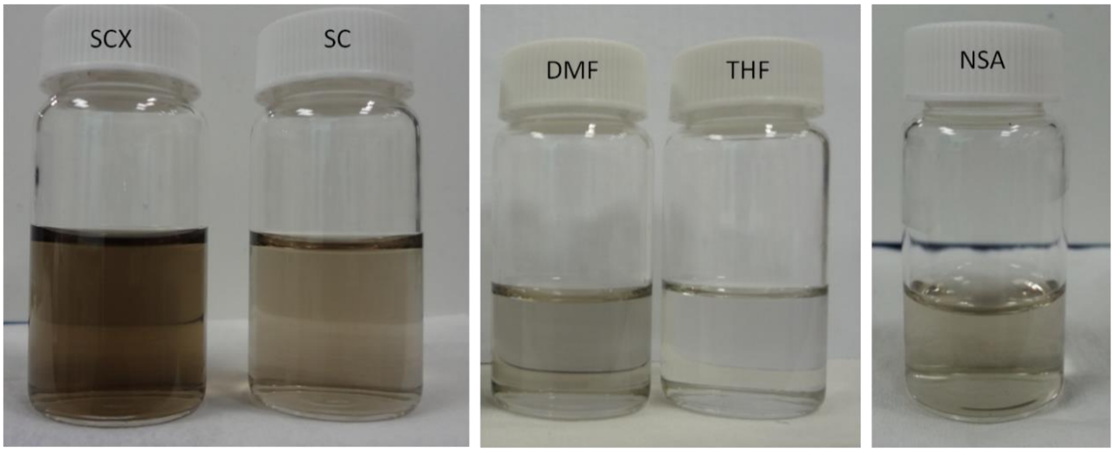

Fig. S4 oGr flakes dispersed in SCX/water (0.5 mM), SC/water (0.5 mM), DMF, THF, and NSA/water.

Notes for preparing the oGr/SCX and oGr/SC dispersion:

oGr flakes (0.5 mg each) were dispersed separately in 0.5 mM SC and 0.5 mM SCX solutions by tip sonication. After tip sonication, both dispersions were centrifuged at 2500 g for 30 mins. The darker color of the oGr/SCX is due to the higher concentration of dispersed/suspended oGr flakes, which illustrates the superiority of SCX over the other surfactants as a dispersion agent for oGr.

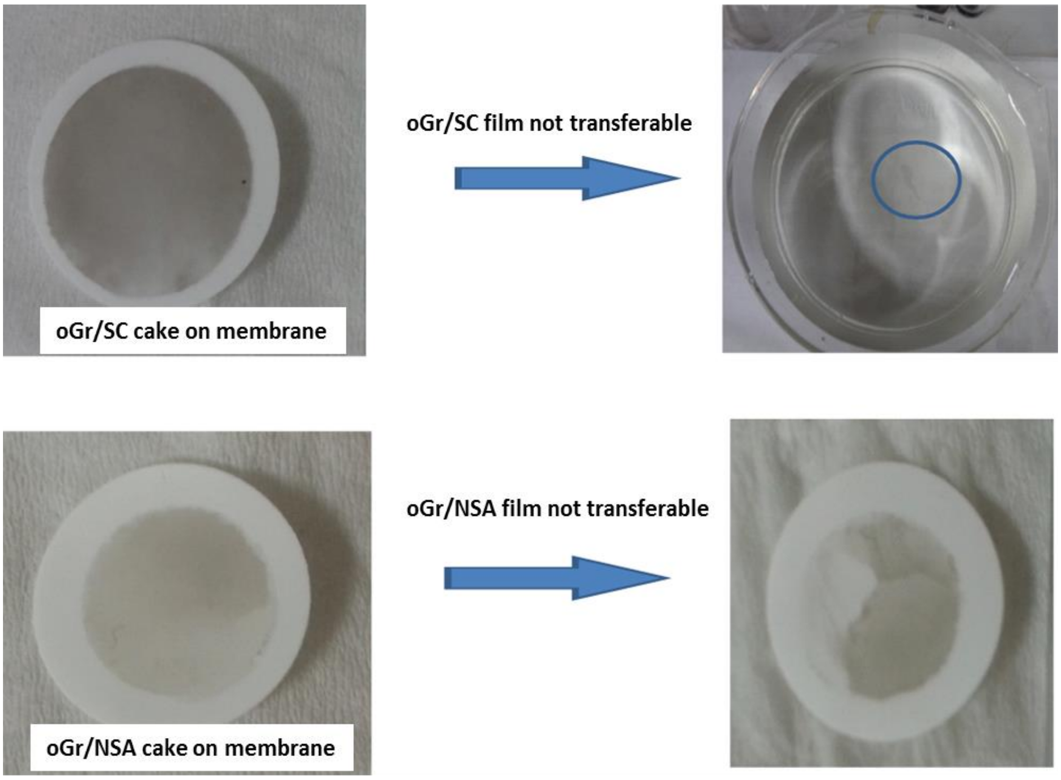

Fig. S5 The oGr film transfer was tried using oGr/SC and oGr/NSA dispersions. Film transfer was unsuccessful due to the failure of a thin film to peel from the wet cake on the filter membrane with SC and NSA dispersing agents.

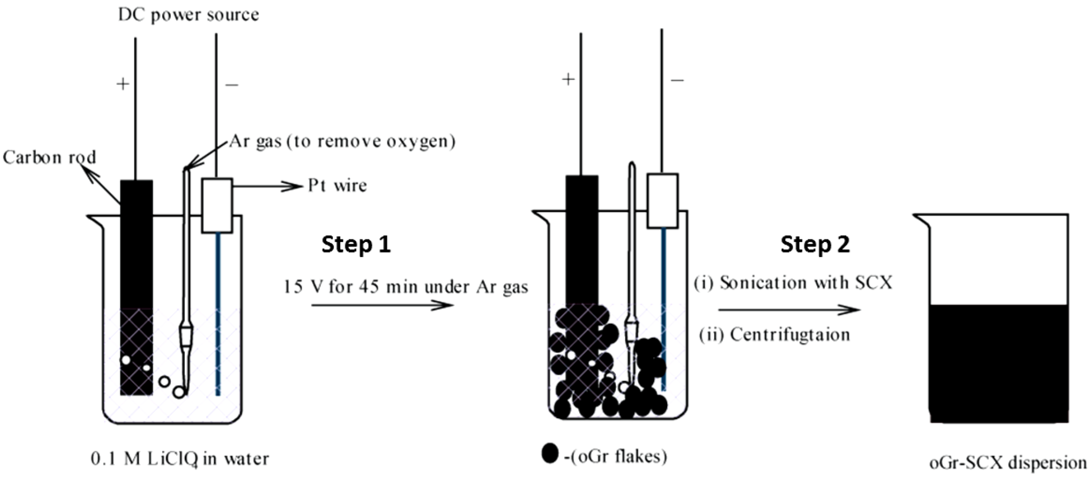

Fig. S6 Process schematic for graphite exfoliation in the presence $LiClO_4$ (0.1 M) under argon environment and preparation of the oGr dispersion in SCX aqueous solution.
